# The establishing of subject positions in Swedish news media discourses during the first year of the COVID-19 pandemic

**DOI:** 10.1177/01634437221147636

**Published:** 2023-01-12

**Authors:** Annica Lövenmark, Jonas Stier, Helena Blomberg

**Affiliations:** Mälardalen University, Sweden; Mälardalen University, Sweden; Mälardalen University, Sweden

**Keywords:** discourse analysis, ideological dilemma, news media, pandemic, subject position, Sweden

## Abstract

The COVID-19 pandemic has dominated the global media since 2020. To a large extent, it is via the news media that the public has learned about the risks, levels of danger, governmental regulations and mandatory actions. This article highlights the subject positions constructed by the Swedish news media from January 2020 to February 2021 in reports about the pandemic. The result shows that citizens can be active-passive or solitary solidarity, these positions appeal to individual accountability, thus potentially shaping and fostering citizens in line with the Swedish government’s wider response to the pandemic. The news media’s images are of self-regulated citizens who govern and discipline themselves and others according to the current discourses, all of which simultaneously evoke fear, togetherness and hope. The ideological dilemmas for citizens are whether to be active-passive or, if necessary, switch to the solitary solidarity subject position.

## Introduction

The first Coronavirus outbreak occurred in Wuhan, China, in December 2019. The virus, COVID-19, spread rapidly throughout the globe and at the end of January 2020 the first case was confirmed in Sweden. At the beginning of February, the Swedish government declared COVID-19 to be a threat to Swedish society (Regeringen, 2020). Within a very short space of time, the Public Health Agency of Sweden (Folkhälsomyndigheten/FHM, 2020) reported a local spread of the virus and in March agreed with WHO’s classification of a pandemic.

During 2020, the COVID-19 pandemic dominated the world’s media. It was via the media that the public were informed about the virus and became aware of the pandemic (Garland and Lilleker, 2021; Kalsnes and Skogerbø, 2021). The media informed the public about the necessary recommendations, regulations and mandatory actions that needed to be taken to prevent the spread of the virus. The media is also one of the many channels that governments (Fairclough, 1995, 2010) use to communicate the public health discourse and individual and collective notions of morals, social behaviour and responsibility (Garland and Lilleker, 2021; Kalsnes and Skogerbø, 2021; Skog and Lundström, 2022).

The Swedish government’s strategy of dealing with the COVID-19 pandemic differed from that of many other countries (Appelgren, 2022; Irwin, 2020). The expert agency, FHM, was responsible for observing and recommending strategies because the Swedish Constitution does not allow for ministerial rule. The development of the pandemic (e.g. restrictions and number of deaths) was communicated via daily press conferences. In these press conferences, FHM, the Swedish Civil Contingencies Agency (MSB, the body responsible for public safety, emergency management etc.) and the National Board of Health and Welfare (Socialstyrelsen) gave updates about the spread of the virus, the number of deaths and intensive care admissions.

The Swedish strategy did not entail a general lockdown, the closing of schools or wearing face masks (WHO, 2020). At the beginning the focus was to control the spread of virus in order to evade overloading the healthcare system. Further into the pandemic, higher education institutions were recommended to move into distance learning and non-essential governmental employees were instructed to work from home. Government representatives often attended the press conferences to stress the seriousness of the development of the pandemic and plead for responsible public behaviour. Stefan Löfven, Sweden’s prime minister, addressed the nation twice and Sweden’s head of state, King Carl XIV Gustav, addressed the nation once, both stressing the importance of ‘common sense’, ‘individual responsibility’, ‘strength’ and ‘self-sacrifice’.

Sweden’s official strategy gained much attention (and criticism) in international media (Appelgren, 2022; Irwin, 2020). Media reported how Sweden differed from other countries, emphasising its unique or even peculiar approach to the pandemic. Irwin (2020) identified six news narratives during the first months of the pandemic: life is normal in Sweden, Sweden has a herd immunity strategy, Sweden is not following expert advice, Sweden is not following WHO’s recommendations, the Swedish approach is failing, and Swedes trust their government. This study showed how the international media constructed and portrayed the pandemic and problematised how media reporting can be misleading.

The media has an impact on launching, legitimising and understanding a problem as well as its solution (Montessori, 2016). In this context, the pandemic is the problem and the vaccine the solution (e.g. Berrocal et al., 2021; Best, 2020; Billig, 2021). The media frames the pandemic by using different rhetorical resources to evoke fear and/or gain disciplinary power over the public, or to hold the public’s attention (e.g. Berrocal et al., 2021; Best, 2020, 2021; Billig, 2021; Craig, 2020; Einboden, 2020; Garland and Lilleker, 2021; Kalsnes and Skogerbø, 2021; Milutinović, 2021; Mohammed et al., 2021; Poulakidakos, 2021). Occasionally, single cases, such as celebrities succumbing to COVID-19, were reported (Best, 2021), although the pandemic was primarily framed and established as a social- and global problem (Berrocal et al., 2021; Best, 2013; Garland and Lilleker, 2021; Kalsnes and Skogerbø, 2021; cf. Spector and Kitsuse, 2009).

There is a need for additional knowledge about how a nation’s media discourses can be used to recruit individuals to engage in collective actions in response to the pandemic (Berrocal et al., 2021; Poulakidakos, 2021). Therefore, a focal question is what subject positions media reports make available for individuals during the pandemic. Here the aim is to highlight the subject positions that are encouraged and constructed by Swedish news media reports about the pandemic between January 2020 and February 2021.

### The news media and the pandemic

How the media framed the pandemic has been studied by several researchers (e.g. Berrocal et al., 2021; Best, 2020; Billig, 2021; Craig, 2020; Einboden, 2020; Milutinović, 2021; Mohammed et al., 2021; Poulakidakos, 2021). Politics, economics and ideologies play an important role in reproducing and legitimising versions of reality (cf. Fairclough, 1995; Van Dijk, 1988, 1998) and has therefore been the focus of studies regarding media discourses on the pandemic. Different rhetorical resources have been used to form the media discourse on the COVID-19 pandemic, such as metaphors (Craig, 2020; Milutinović, 2021; Semino, 2021), the politics of how to label the pandemic (Berrocal et al., 2021; Poulakidakos, 2021), numbers and/or statistics (Best, 2020, 2021; Billig, 2021), photographs of citizens in different age groups (Martikainen and Sakki, 2021) and the rhetorical use of nurses as a specific category (Boulton et al., 2022; Einboden, 2020; Mohammed et al., 2021). Nurses on the COVID-19 frontline have been framed as ‘heroines’ or ‘heroes’. Such a ‘discourse’ positions nurses as productive subjects and influences how the public should think and act during the pandemic. The heroic discourse is a tool with which politicians and stakeholders can express support for the nurses in the media to gain disciplinary power over the public (Boulton et al., 2022; Mohammed et al., 2021). The media’s use of citizen’s photographs to portray COVID-19 constructs both the social representations and identities of groups (Martikainen and Sakki, 2021). Within the discourse, in-groups and out-groups are formed, based on and expressed by individuals’ motivations, ideologies, self-categorisations, actions and shared values (Berrocal et al., 2021). Martikainen and Sakki’s (2021) visual rhetoric analysis reveals several subject positions related to age, for example, adults as authoritative experts, adaptive professionals, responsible caretakers or active recreationist. Occupying a subject position as an individual can evoke ideological dilemmas when negotiating identity, for example, vegans struggling with taken-for-granted-knowledge about how eating meat is a hegemonic practice (Buttny and Kinefuchi, 2020).

The rhetorical use of ‘large round numbers’, such as ‘thousands’ and statistics in the news media, evokes fear and a sense that the pandemic concerns us all (Billig, 2021). Counts and cross-national comparisons suggest that the numbers of COVID-19 infections and deaths are underestimated (Best, 2020). Also war and military metaphors have been frequently used in the media coverage of the pandemic (Berrocal et al., 2021; Craig, 2020). Sontag’s (1979) study of illness as a metaphor has inspired research on the use of metaphorical expressions in communication, for example, in media discourses on viruses before COVID-19 (Vasterman and Ruigrok, 2013; Wallis and Nerlich, 2005) and during it (Abdel-Raheem, 2021; Craig, 2020; Milutinović, 2021; Panzeri et al., 2021; Semino, 2021). Studies of political cartoons show that war metaphors are often used in a social and political way to make sense of situations. Abdel-Raheem (2021) suggests that the pandemic itself has become a metaphor and that the very term pandemic can convey fear of other things as well.

There is a knowledge gap in the previous research of news media discourses about the pandemic concerning how the news media produces national images of how the public should act, think and talk (Foucault, 1980/2010; Rose, 1999) about the pandemic. Even though some studies emphasise how politicians can use media to gain disciplinary power over the public (Berrocal et al., 2021; Boulton et al., 2022; Mohammed et al., 2021; Poulakidakos, 2021) or shows possible subject positions due to age (Martikainen and Sakki, 2021) there is a need for knowledge about possible subject positions in news media discourses. Here the focus is on news media discourses on the pandemic in a Swedish context. When studying the use of language in the media reports possible subject positions can be identified. By illustrating how Swedish citizens are encouraged to adopt certain subject positions, the analysis shows how the public can govern themselves by the news media’s discourses.

## Rationale, aim and research questions

Due to the knowledge gap the ambition with this article is to contribute to how the Swedish news media, as a governmentality function, fosters and appeals to citizens during the pandemic. By focussing on Sweden as an example and the use of language in the news media’s reports of the pandemic, this study identifies and discusses the possible subject positions that Swedish citizens can adopt, negotiate or reject. The results shed light on the Swedish news media’s discourses, what is taken for granted and how they establish certain ways of thinking, talking and acting towards the pandemic. The following questions are addressed in the study:

1. How and what kind of rhetorical resources are used to establish subject positions?
2. Which subject positions can be identified in the Swedish news media?
3. Are there any clashes between or within these subject positions?

The results contribute to knowledge about the discourses on the pandemic in a national context and the possible subject positions that are adopted in relation to global discourses on the pandemic. Moreover, the results show how individuals submit themselves to the power of the news media and thereby become self-regulated, governed objects acting within and consistent with the established discourses.

## Theoretical framework and research method

In order to identify what is conveyed and discursively constructed in the news media, this study draws on governmentality (Foucault, 1994; Rose, 1999) and a rhetorical approach (Potter, 1996; Potter and Edwards, 1992; Potter and Wetherell, 2001; Semino, 2008). Governmentality are conceptualised as power and authority relations which affect the identities of individuals. Governmentality presupposes individual freedom and assumes that individuals help co-create their own governance and is characterised by a relation between actions considered desirable and the responsibility of the individual to act in accordance with them. Language is the foundation for governance in terms of who can speak, what is true and what positions are used to speak; discourses (Foucault, 1994; Rose, 1999).

Continuous news feeds provide us with media-generated images of the world that are then used to construct and make sense of our everyday lives. Thus, the media both co-constructs the reality and has the power to set the agenda (Billig, 1987/1996; Fairclough, 1995). Journalists and editors do this by using discourses that should and can be told in each context (Fairclough, 1995, 2010; Foucault, 1980, 2010; Rose, 1999). Analysing media discourses involves identifying the structure of the news media, who is entitled to speak and how and which ideologies have become naturalised and part of ‘common sense’ (Fairclough, 1995; Foucault, 1980, 2010; Van Dijk, 1988, 1998). In the case of the pandemic, the news media is a powerful agent for constructing a common understanding of ourselves as subjects (Milutinović, 2021). Discourses constitute, establish and reproduce the physical world, individuals as subjects, power relations and their effects (Foucault, 2003; Rose, 1999). In turn, discourses are constructed through language. Thus, in order to see how discourses affect reality, it is necessary to see how language affects discourses. This can be done by analysing the media’s narrative rhetoric.

In a rhetorical analysis of language-use the following rhetorical resources are important: (1) extreme case formulations for legitimising truth claims by down-sizing, and/or exaggerating and/or repeating phrases such as: all of us, very urgent, acting powerfully (Pomerantz, 1986; Potter, 1996), (2) the pronoun we for launching and establishing a collective or to distance our own accountability. It also establishes the authority of government and experts as a knowledgeable ‘we’ (Potter, 1996; Potter and Wetherell, 2001), (3) time to claim that events have actually happened or to emphasise speed or lack of speed in a situation (Potter, 1996) and (4) metaphors for persuading, conveying and emphasising a statement/feeling/situation (Lakoff and Johnson, 1980/2003; Semino, 2008). When analysing language used in news media, it is possible to show how categories and discourses can establish and produce power, knowledge, values and truths about the world and the individuals inhabiting it (Hall, 2001; Potter, 1996). For example, being a parent is a socially constructed category and commonly regarded as something ‘natural’ and is therefore often taken for granted. In relation to different discourses, this category can be rejected, negotiated or regarded as true (Hall, 2001; Potter, 1996).

Socially constructed categories are products of discourses and situated in context, culture, time and place, which regulates what is possible or not possible to say, do and feel (Foucault, 2003; Rose, 1999). A specific category can entitle an individual to certain actions or expertise (Potter, 1996). One or more person categories (e.g. mother or father) and their categorised actions (e.g. feed or foster) constitute a subject position – a concept of identity and the self that is made possible through language. A subject position is constructed and enabled by which person categories and categorised actions an individual regards as logical, proper and significant (Billig, 1987/1996; Hall, 2001). A subject position is thus created and made possible by social categories, and the person categories and categorised actions that are meaningful and logical for an individual in relation to others (Billig, 1987/1996; Edley, 2001; Hall, 2001).

The concept of ideological dilemma (etic) (Billig, 1988, 2001) can be used to explore and describe different and competing views of what is regarded as common sense. By distinguishing cognitively constructed intellectual ideologies from lived ideologies, the latter are considered to be constructed on observations, values and language in a specific social context. One example of an ideological dilemmas is a firm attitude versus mercy, which are valued according to the underlying logic. Discourses can be regarded as practical ideologies, since their function becomes visible when they make sensible experiences meaningful. These reason-based discursive practices are ideological in that they support certain ideas or thought structures. The ideological dilemma can be described as different or competing versions of what is considered as common sense in a situation or action and is not to be taken for granted (Billig, 2001).

The analytical process focused on how the text was rhetorically organised (Hall, 2001; Lakoff and Johnson, 1980/2003; Pomerantz, 1986; Potter, 1996; Potter and Wetherell, 2001; Semino, 2008). The identification of subject positions (e.g. active citizen) was conducted through the classification of person categories (e.g. the majority) and categorised actions (e.g. understanding or learning). The concept of ideological dilemma was used to capture possible clashes between or within a subject position. The uniform interpretations of etic and emic are presented as subject positions, including their ideological dilemmas. Table 1 illustrates the analysis process:

**Table 1.** Example of the analysis process.

Quote	Code subcode	Person category	Categorised action	Subject position	Ideological dilemma
‘The majority understand how important vaccines are’ Kudo, P. (02-02-2020)	Active(etic) Understand (emic)	The majority (emic)	Understand (emic)	Active (etic)	The active-passive citizen (etic)

## Empirical material and methodological reflections

The data was collected from the Swedish Retriever Research Database (www.retriever.se) based on the words pandemic and vaccine, where the pandemic was framed as the problem and vaccine as the solution (cf. Montessori, 2016) during the first year of the pandemic in a Swedish context (January 2020–February 2021). The search resulted in 4000 hits. Articles, editorials, debate posts and reports from five of Sweden’s largest daily newspapers (Aftonbladet, Dagens Nyheter, Göteborgs-Posten, TT Nyhetsbyrån and Svenska Dagbladet) were also included in the data, resulting in a match of 376 articles. After a thorough reading of the titles and content, 237 articles were included in the data collection. The inclusion criteria for these 237 articles were descriptions of a person category or categorised action for Swedish citizens, the pandemic and vaccinations.

The authors followed the ‘Ethical international standards for responsible research publication for authors’ (Wager and Kleinert, 2011). Interpreting and giving other people’s words and stories meaning relate to validity, ethics and power (Potter, 1996; Schegloff, 1997; Wetherell, 1998). Reflexivity means accounting for how and what kind of knowledge re-searchers create (Potter, 1996). There are always layers of context to consider, for example, Sweden as the actual place, the news media’s logic for presenting newsworthy reports, the media narratives’ context and analysing what is directly and indirectly conveyed (Bell, 2001). The reflexive approach to the construction of data includes an emic and etic stance (Headland et al., 1990; Pike, 1993; Silverman, 1993). The explicit (emic) person categories (e.g. we) and categorised actions (e.g. need) in the text and the analytically formed (etic) person categories (e.g. citizen) and categorised actions (e.g. passive) were organised into sub-codes and codes using the software programme NVivo, in order to search for common features and content. These person categories and categorised actions were then combined and abstracted into analytically created subject positions (etic) (Edley, 2001; Reynolds and Wetherell, 2003). The next step was to analyse possible conflicts among or within the constructed subject positions and formulate subject positions representing the identified ideological dilemma (Billig, 2001). The analysis shows which subject positions and discourses the news media established for Swedish citizens in the context of the pandemic and vaccines.

News media feeds generate a specific and chronological flow of images of the pandemic and vaccines. To illustrate this feed, several quotations are presented together in one and the same example of the empirical findings.

## Media content analysis results

In February 2020, the Swedish news media stated that the pandemic was a global issue and was likely to threaten the nation (Berlid Lundblad, 2020; Pirttisalo Sallinen and Kudo, 2020). In the spring of that year, the news media portrayed a nation at war against an invisible enemy that would cause global economic collapse, destruction and death (Hansson, 2020). The pandemic (virus) was portrayed as the nation’s ‘problem’ (Foucault, 1990; Rose, 1999) and the available vaccines as the ‘solution’ (Foucault, 1990; Rose, 1999). Feelings like fear of the virus and vaccines as the hope for salvation were resources that were supposed to invoke people’s desirable behaviour (Foucault, 1990, 1994). Citizens were expected to willingly self-regulate, make themselves aware of the ongoing and accumulated knowledge (Foucault, 1990, 1994) of the government and its agencies and trust their expertise; governmentality. (Rose, 1999).

### Rhetorical features in the news media’s construction of subject positions

In order to establish a common understanding of the kind of subjects the government expected to evoke, the rhetorical use of the pronoun we were frequently repeated in combination with time, extreme case formulations (Pomerantz, 1986; Potter, 1996) and metaphors (Lakoff and Johnson, 1980/2003; Semino, 2008). Common formulations in the news media were:

We must intensify our actions (Nilsson, 2020) . . .//. . . we need to act forcibly if we are to control the spread of the virus . . .//. . .we should act swiftly now to keep the virus in check and not wait until tomorrow (Göteborgs-Posten, 2020) . . .//. . . we have started for too late and need to catch up (Hanson, 2020) . . .//. . . we need to join together to confront the common enemy (Lagercrantz, 2020). . . we are following the situation minute by minute, hour by hour in order to be able to take the necessary actions (Hjörne, 2020a)

When an expert in the field of crises in the EU states ‘we must intensify. . .’ (Nilsson, 2020), the use of we convey a feeling of togetherness for Swedish citizens as a nation as well as a part of Europe. This can support each person’s direct actions and, at the same time, appeal to everyone to act as a united body. Likewise the use of ‘we need to act’ in headlines (Göteborgs-Posten, 2020) the news media calls directly to each citizen as individuals as well as a collective. The quotes above also illustrates how news media continues to use ‘we’ in order to strengthen the importance of togetherness in the nation (Hanson, 2020; Lagercrantz, 2020).

In another news media report, when the prime minister states: ‘we are following the situation minute by minute, hour by hour in order to be able to take the necessary actions’ (Hjörne, 2020a) the government presents itself as a united knowledgeable we and appeals to the general Swedish public to follow as a collective we. Time reinforces the importance of acting quickly. Here, the government thinks and decides for the citizens. Time also creates a factual reality that everyone can acknowledge and handle (Potter, 1996). Rhetorically, the use of extreme case formulations (‘minute by minute’, ‘act forcibly’) by the news media support the imperative to take action. It is important, it has to happen swiftly, and everybody needs to follow the lead, the thinking and the acting. Metaphors in the above quote are for example, that of a ticking clock (minute by minute, hour by hour) and the use of war (common enemy, intensify), which reinforce the effects created by the other rhetorical devices (we, time). At the same time, the metaphors make the message more appealing and create a visible image of a nation in great danger and the joint need for important, quick, united action.

### Being an active-passive citizen

At the end of February 2020, the news media prepared citizens for the fact that a severe global pandemic had arrived. Here, individuals were expected to govern themselves by actively display actions such as understanding, knowing and learning. In the news media people were frequently asked to be prepared by keeping a distance and isolating:

The majority understand how important vaccines are (Kudo, 2020) . . .//. . .the vast majority take all this very seriously (Letmark and Dahl, 2020) . . .//. . .Not everyone has understood this yet, but it is important that we all take responsibility and play our part in order to maintain the current momentum and minimise direct contact with others . . .//. . . All those who can work from home should do so. Everyone should avoid gatherings of more than a few people. Every single person should minimise their contact with the health services in order to keep the pressure to a minimum (Ludvigsson, 2020)

Here, the media images try to convey to the majority of Swedish citizens the importance of understanding the need for vaccines, the seriousness of the situation and the need to act together. This is accomplished by using ‘extreme case formulations, such as minimising, in combination with the pronouns we and they and phrases like the majority, the vast majority, all, everyone and every single person. The collective act is sanctioned when each individual is required to learn about the pandemic and how to handle it. The actions to minimise interactions with others carry an ideological dilemma (Billig, 1987/1996), that is, being active and acting, even though at the same time the required actions lead to a passive state and varying degrees of isolation for everybody. Another example of being active and also passive is the formulation maintain the current momentum and minimise direct contact with others.

In March and April 2020, some weeks after the pandemic was confirmed in Sweden, the news media portrayed the situation as highly insecure and frightening. In this context, the use of ‘we’ direct the messages to Swedish citizens and demand them to search for more knowledge from the government’s public health experts. At the same time the use of ‘we’ strengthen the authority of the experts as a knowledgeable ‘we’:

We look to those who know how we can get help to survive (Sachs, 2020) . . .//. . .General knowledge about how to protect oneself and others from the virus has an effect (Manzoor, 2020a) . . .//. . . Now we know that the virus can spread quickly across the world and put a strain on the health services (Manzoor, 2020b) . . .//. . .We know, but we don’t act until it is too late. Not only that, we scale down our preparedness against our better judgement (Söderqvist, 2020)

Thus, due to an ongoing need for knowledge and learning, citizens are encouraged to act in accordance with the news media discourse and discipline and govern themselves as subjects. Submitting to this power and knowledge means taking disciplinary action (Foucault, 1990, 1994; Rose, 1999) so that the collective can protect itself. The news media confirms and monitors that these actions are effective, despite being insufficient. The news media legitimises the required actions given that the pandemic is a global issue and communicates that a collective sense of war is necessary for survival. The use of the war metaphor in the above quote indirectly portrays a nation that is suddenly under attack from the enemy without knowledge or the readiness to fight back. From a retrospective perspective, the acquired knowledge is at hand, but it is too late because our preparedness is already scaled down against our better judgement.

The actions of being prepared, learning, accepting, getting used to, fighting, social distancing, washing hands and isolating are frequently proclaimed and emphasised in the news media. Also in this context, the use of ‘we’ speaks directly to Swedish citizens for required actions and be humble and know that citizens as well as experts has to constantly learn and grow in knowledge:

We need to constantly learn as much as possible about COVID-19 because we will have to live with the virus in the foreseeable future, even if vaccines become available (Berglöf, 2020) . . .//. . . In short, we will have to learn to live with a certain level of infection and get used to washing our hands and surfaces and maintaining social distance (Von Hall and Widell, 2020) . . .//. . . Vaccinations will be a serious and decisive step towards combatting this terrible pandemic. But the danger is far from over, we must continue to keep a distance, stay at home and only fraternise with those in the same household (Magnusson and Eliasson, 2020) . . .//. . . We must be ready to fight back against new outbreaks. Until we have a vaccine, social distancing is the only thing we have (Von Hall, 2020)

Here, active citizens are expected to be constant learning subjects and demonstrate self-regulated and well governed behaviour (Foucault, 1990, 1994; Rose, 1999). In order to govern oneself and others, people are required to be active by for example, washing their hands and social distancing. At the same time, they are required to be passive and willingly absorb the facts and actions that the experts state as true and necessary. The actions of understanding, knowing, learning, being prepared, distancing and isolating create the ideological dilemma (Billig, 1987/1996) of being a citizen that actively follows yet passively submits to these truths and regulations. The war metaphor reinforces the seriousness of the situation and the need for quick and armed actions: We must be ready to fight back against new outbreaks. The action of social distancing is further legitimised by the single weapon at hand. The media generated images define and limit what is to be regarded as real and true. The establishment and reproduction of this media discourse on the pandemic and vaccines are supposed to be taken for granted and acted on by all citizens.

### Being a solitary solidarity citizen

Another subject position produced by the news media is the solitary solidarity citizen. The use of the war metaphor recurs when citing WHO and requiring all citizens to act as solidarity global citizens. The use of ‘we’ are resourceful in requiting individuals to this subject position:

Global solidarity is required. . .//. . .The countries of the world openly collaborate with each other and create a united front in our efforts to get the situation under control (Pedersen, 2020) . . .//. . .Yes, we also work together to confront the common enemy. In my view, the basic qualities that we humans have been schooled in are wonderful strengths that we can take advantage of and trust if we are to deal with the pandemic effectively (Lagercrantz, 2020) . . .//. . . Many of us are always thinking of relations who are older or in some way vulnerable. Those for whom what we call social readiness is mainly for. Now, “otherliness” is the worst imaginable companion. Everyone without exception has a responsibility to prevent the virus from spreading. This is both the only and best thing we can do (Ludvigsson, 2020)

Thus, the need for global and national solidarity in this war-like situation is established by using words like cooperation, collaboration, working together and confronting a common enemy. The rhetorical use of war metaphors strengthens the serious condition that all citizens need to be aware of. It also implies the urgent need to act on behalf of others who may be unable to take similar actions, such as the elderly or the vulnerable. The quality of an individual and collective solidarity is regarded as an effective and trustworthy human strength for nations at war. The only and best thing we can do is to be individually responsible, which is a necessary altruistic act. The news media rhetoric invokes the sense of individual accountability to join a national and global collective to protect another collective (the elderly and vulnerable). The solitary solidarity subject position dilemma is constructed when solidarity means isolation and at the same time subordinating to a news media constructed national or global collective. In other words, citizens are forcibly subordinated to a media constructed collective, where not being an accountable citizen in unthinkable.

In the summer months citizens were reminded of the need to act to keep up and learn to live with the required actions and regulations. Even if a vaccine becomes available in the future, the solidarity citizen should not abandon this recommended individual and collective governed behaviour:

But we need to realise that the virus is here to stay, even well after we have a vaccine. We must learn to live with it. This means that we cannot relax, but must continue to wash our hands and keep a distance (Manzoor, 2020c)

In November 2020, Swedish citizens were assured that everyone would need to be vaccinated. The prime minister was not satisfied with Swedish citizens’ responses so far. The solidarity action of showing respect had declined and, in order to reverse this trend, the number of deaths was used as a rhetorical resource (Potter, 1996):

At present the rate of infection is alarmingly high and resulting in yet another wave of deaths. Sweden has reported 559 deaths in the last two weeks (Wolodarski, 2020) . . .//. . . The respect that Swedish citizens showed in spring is not visible to the same extent, which is not good enough in a pan-demic where more than six thousand people in our country have died. A total and real change in behaviour is now imperative, says the prime minister . . .//. . .Everyone living in Sweden now has a grave responsibility. Each and every one of us needs to do our duty and take responsibility for stopping the spread of this virus. We all have a responsibility to find out which advice and recommendation are valid in the areas where we live (Karlén, 2020)

To act in solidarity, citizens are asked to radically change their behaviour, take grave responsibility and do their duty. To fulfil the requirements of accountability citizens are expected to individually know and act on the local and national collective regulations. Extreme case formulations (Pomerantz, 1986) such as heavy, grave, all and each and every one of us, reinforce the message to act now in accordance with the appealed for individual governmentality and accountability (Foucault, 1990, 1994; Rose, 1999).

## Discussion

The aim of this article has been to contribute new knowledge about how Swedish news media discourses were used to recruit governed individuals during the first year of the COVID-19 pandemic. Additionally, it has identified the subject positions that Swedish citizen were asked to occupy in this situation. The findings suggest that during the studied period the Swedish news media discourses produced two dominant subject positions for citizens to occupy or negotiate in the endeavour to understand and manage the pandemic. One is that the pandemic is framed as a global and a national social problem (cf. Berrocal et al., 2021; Montessori, 2016). The other is vaccine as the desirable solution across the globe (cf. Best, 2020, 2021).

These subject positions come into play by appealing to feelings, such as fear of the virus and vaccine as the hope for salvation. Feelings are frequently used in Swedish news media narratives to evoke citizens’ desirable behaviour according to a pattern similar to that in for example UK, Canada and Norway (cf. Abdel-Raheem, 2021; Billig, 2021; Garland and Lilleker, 2021; Kalsnes and Skogerbø, 2021). The discourses are effectively based on citizens’ self-regulation and willingness to constantly search for more knowledge and to trust the appointed national experts and authorities (Foucault, 1990, 1994; Rose, 1999). By using the available ideologies about the pandemic as the problem and vaccine as the solution, the news media discourses establish a common understanding of the pandemic for all citizens (cf. Fairclough, 1995; Van Dijk, 1988, 1998).

Regarding which rhetorical resources are used to establish these subject positions, a key finding is the use of the pronoun we, time (Potter, 1996), extreme case formulations (Pomerantz, 1986) and metaphors (Lakoff and Johnson, 1980/2003; Semino, 2008). These rhetorical resources naturalise the communicated ideologies. When authorities, experts and stakeholders construct a collective we as knowledgeable and trustworthy, citizens are expected to both comply with and respond as part of that ‘we’. One result is that a feeling of reasonable unity to accept and act on is constructed and associated with the nation as such, which is in line with previous international research (cf. Berrocal et al., 2021; Craig, 2020; Garland and Lilleker, 2021; Kalsnes and Skogerbø, 2021; Poulakidakos, 2021). The use of time in combination with extreme case formulations and metaphors reinforces the urge to act as a unified nation and the importance of acting swiftly and in compliance with what is regarded as important and true. In relation to other studies (Best, 2020, 2021; Billig, 2021), the use of numbers of deaths evoke fear and citizens are encouraged to understand the situation as severe and act solidary.

A second key finding is the two subject positions and clashes between and within these subject positions. To fulfil the nation’s need for citizens to act, based on the common ‘we’ in this time of crisis, the news media portrays two possible subject positions for Swedish citizens: the active-passive citizen and the solitary solidarity citizen (cf. Berrocal et al., 2021; Milutinović, 2021; Skog and Lundström, 2022). The image created by the news media is a somewhat homogeneous picture of the kind of subjects that are required by the nation in a time of crisis (cf. Garland and Lilleker, 2021; Kalsnes and Skogerbø, 2021). This can be compared to the visual rhetorical analysis of Martikainen and Sakki (2021), which identifies several heterogenic subject positions. The reason for the homogenic findings could be the media’s image of the stakeholders’ mission to govern citizens into accountability or the result of this study’s limitations, that is, the selection/inclusion criteria and the sole use of mainstream news media. Nevertheless, in the first year of the pandemic the news media did not enable citizens to create their own or different subject positions. In some cases, the news media reported on individual’s undesirable behaviour of not complying with the stakeholders’ and the media’s agendas (cf. Berrocal et al., 2021; Skog and Lundström, 2022). Rather than opening up for the possibility to occupy other positions, the message communicated by the media was to establish them as deterrent examples in order to avoid blame.

The subject position for the active-passive citizen requires specific actions and skills, for example, understanding, knowing and learning. This subject position carries an ideological dilemma (Billig, 1987/1996; cf. 2001) of whether to be active and act. At the same time, the required actions lead to a passive and varying degree of isolation for all. The dilemma is that subjects need to switch between being active and passive in what is described by the news media as a highly insecure and scary situation. By requiring citizens to adopt a subject position, the news media asks them to submit to the power, knowledge and disciplinary system promoted by the stakeholders (cf. Berrocal et al., 2021; Boulton et al., 2022). This subject position is also constructed on the basis of an existing war metaphor for the pandemic, for example, being prepared, getting used to the situation and fighting the enemy.

When the active-passive subject position does not produce enough active-passive citizens with the desirable self-regulated behaviour the news media discourse on the pandemic as a war (cf. Abdel-Raheem, 2021; Berrocal et al., 2021; Craig, 2020; Milutinović, 2021; Semino, 2021) appeals to them to occupy yet another subject position - that of a solitary solidarity citizen (cf. Berrocal et al., 2021). The main task for citizens is to do their duty, submit to the powers that be and act in solidarity in a national and global crisis. The requirement here is to isolate and at the same time become part of an unknown global and national collective; the solidary ‘in-group’ (cf. Berrocal et al., 2021; Skog and Lundström, 2022) the news media has created. Nonetheless, as shown in this article, the news media has an established and widespread social function of shaping public opinion on behalf of political-, economic- and ideological interests (cf. Fairclough, 1995; Garland and Lilleker, 2021; Kalsnes and Skogerbø, 2021; Milutinović, 2021; Van Dijk, 1988, 1998) which have the power to spread, legitimise and reproduce specific versions of reality (cf. Billig, 1999; Montessori, 2016).

## Conclusion

The central ideological dilemma in the news media reports on the COVID-19 pandemic concerns how to deal with a new, uncertain and at times frightening life situation based on the requested and available ‘in-group’ and moral subject positions of being an active-passive citizen or a solitary citizen in solidarity. The findings show how the news media appeals to citizens individual accountability and responsibility, thus potentially shaping and fostering them in line with the Swedish government wider response to COVID-19. In order to communicate this, the news media produces images of self-regulated citizens who govern and discipline themselves and others according to the current news media discourses of fear, togetherness and hope.

## References

[bibr1-01634437221147636] Abdel-Raheem A (2021) Reality bites: How the pandemic has begun to shape the way we, metaphorically, see the world. Discourse & Society 32(5): 519–541.

[bibr2-01634437221147636] Aftonbladet (2020) Ta rygg på oss äldre, ungdomar. Aftonbladet, 19 November, 20.

[bibr3-01634437221147636] Appelgren E (2022) Media management during COVID-19: Behavior of Swedish media leaders in times of crisis. Journalism Studies 23(5-6): 722–739.

[bibr4-01634437221147636] Bell A (2001) The discourse structure of news stories. In: Bell A Garrett P (eds) Approaches to Media Discourse. Oxford: Blackwell Publishers, pp.64–104.

[bibr5-01634437221147636] Berglöf E (2020) Forskarna har lärt av krisen - men inte beslutsfattarna. Dagens Nyheter, 2 June, 7.

[bibr6-01634437221147636] Berlid Lundblad N (2020) Förr eller senare drabbas vi alla av epidemierna. Svenska Dagbladet, 18 January, 28.

[bibr7-01634437221147636] Berrocal M Kranert M Attolino P , et al. (2021) Correction: Constructing collective identities and solidarity in premiers’ early speeches on COVID-19: A global perspective. Humanities & Social Sciences Communications 8: 158. 34190730 10.1057/s41599-021-00841-7 PMC8223763

[bibr8-01634437221147636] Best J (2013) Social Problem. New York, NY: W.W. Norton & Com.

[bibr9-01634437221147636] Best J (2020) COVID-19 and numeracy: How about them numbers? Scholar Commons 13(2): 1–17.

[bibr10-01634437221147636] Best J (2021) How to lie with coronavirus statistics: Campbell’s law and measuring the effects of COVID-19. Scholar Commons 14(1): 1–17.

[bibr11-01634437221147636] Billig M (1987/1996) Arguing and Thinking: A Rhetorical Approach to Social Psychology. Cambridge: Cambridge University Press.

[bibr12-01634437221147636] Billig M (1988) Ideological Dilemmas: A Social Psychology of Everyday Thinking. London: SAGE.

[bibr13-01634437221147636] Billig M (1999) Whose terms? Whose ordinariness? Rhetoric and ideology in conversation analysis. Discourse & Society 10(4): 543–583.

[bibr14-01634437221147636] Billig M (2021) Rhetorical uses of precise numbers and semi-magical round numbers in political discourse about COVID-19: Examples from the government of the United Kingdom. Discourse & Society 32(5): 542–558.

[bibr15-01634437221147636] Billig MJ (2001) Discursive, rhetorical and ideological messages. In: Wetherell M Taylor S Yates S (eds) Discourse Theory and Practice: A Reader. London: SAGE, pp.210–221.

[bibr16-01634437221147636] Boulton M Garnett A Webster F (2022) A Foucauldian discourse analysis of media reporting on the nurse-as-hero during COVID-19. Nursing Inquiry 29: 1–12. 10.1111/nin.12471 PMC8646255 34729856

[bibr17-01634437221147636] Buttny R Kinefuchi E (2020) Vegans’ problem stories: Negotiating vegan identity in dealing with omnivores. Discourse & Society 31(6): 565–583.

[bibr18-01634437221147636] Craig D (2020) Pandemic and its metaphors: Sontag revisited in the COVID-19 era. European Journal of Cultural Studies 23(6): 1025–1032.

[bibr19-01634437221147636] Edley N J (2001) Analyzing masculinity: Interpretative repertoires, ideological dilemmas and subject positions. In: Wetherell M Taylor S Yates S (eds) Discourse as Data: A Guide for Analysis. London: SAGE, pp.189–228.

[bibr20-01634437221147636] Einboden R (2020) SuperNurse? Troubling the Hero Discourse in COVID Times. Health: An Interdisciplinary Journal for the Social Study of Health Illness and Medicine 24(4): 343–347. 10.1177/1363459320934280 32538181

[bibr21-01634437221147636] Fairclough N (1995) Media Discourse. London: Edward Arnold.

[bibr22-01634437221147636] Fairclough N (2010) Critical Discourse Analysis. The Critical Study of Language. Harlow: Longman.

[bibr23-01634437221147636] FHM (Folkhälsomyndigheten) (2020) Förslag: Ytterligare begränsningar av allmänna sammankomster [In English: Suggestion: Additional restrictions for public gatherings], March 27. Available at: https://www.folkhalsomyndigheten.se/nyheter-och-press/nyhetsarkiv/2020/mars/forslag-ytterligare-begransningar-av-allmanna-sammankomster/ (accessed 16 March 2021).

[bibr24-01634437221147636] Foucault M (1980/2010) Power/Knowledge: Selected Interviews & Other Writings 1972-1977. New York: Phantheon Books.

[bibr25-01634437221147636] Foucault M (1990) The history of sexuality Vol. 1 The will to knowledge. Harmondsworth: Penguin.

[bibr26-01634437221147636] Foucault M (1994) Essential Works of Foucault 1954-1984, Volume 3, Power. Harmondsworth: Penguin.

[bibr27-01634437221147636] Foucault M (2003[1973]) The Birth of the Clinic, an Archaeology of Medical Perception. London: Routledge.

[bibr28-01634437221147636] Garland R Lilleker D (2021) The UK: From consensus to confusion. In: Lilleker D Coman I Gregor A , et al. (eds) Political Communication and COVID-19. Governance and Rhetoric in Times of Crisis. London: Routledge, pp.165–176.

[bibr29-01634437221147636] Göteborgs-Posten (2020) Vi måste agera kraftfullt för att få kontroll på smittspridningen. Göteborgs-Posten, 1 April, 5.

[bibr30-01634437221147636] Hall S J (2001) Foucault: Power, knowledge and discourse. In: Wetherell M Taylor S Yates S (eds) Discourse Theory and Practice: A Reader. London: SAGE, pp.72–81.

[bibr31-01634437221147636] Hanson S (2020) Inför striktare regler i tre till fyra veckor. Dagens Nyheter, 15 April, 18.

[bibr32-01634437221147636] Hansson W (2020) Tiden börjar rinna ut för att stoppa smittan. Aftonbladet, 25 February, 7.

[bibr33-01634437221147636] Headland TN Pike KL Harris M (1990) Emics and Etics: The Insider/Outsider Debate. London: SAGE.

[bibr34-01634437221147636] Hjörne P (2020a) Beredskap, kanske - men förmåga? Göteborgs-Posten, 1 March, 2.

[bibr35-01634437221147636] Irwin RE (2020) Misinformation and de-contextualization: international media reporting on Sweden and COVID-19. Globalization and Health 16(1): 62–12. 10.1186/s12992-020-00588-x PMC7356107 32660503

[bibr36-01634437221147636] Kalsnes B Skogerbø E (2021) Norway: From strict measures to pragmatic flexibility. In: Lilleker D Coman I Gregor A , et al. (eds) Political Communication and COVID-19. Governance and Rhetoric in Times of Crisis. London: Routledge, pp.231–238.

[bibr37-01634437221147636] Karlén M (2020) Sverige har säkrat tillgången på vaccindoser för alla. Göteborgs-Posten, 18 November, 8.

[bibr38-01634437221147636] Kudo P (2020) Svenska forskare jobbar för att ta fram vaccin mot coronaviruset. Svenska Dagbladet, 2 February, 9.

[bibr39-01634437221147636] Lagercrantz A (2020) Yaleprofessor: Vår “sociala kostym” är räddningen ur krisen. Svenska Dagbladet, 26 April, 28.

[bibr40-01634437221147636] Lakoff G Johnson M (1980/2003) Metaphors We Live by. Chicago, IL: The University of Chicago Press.

[bibr41-01634437221147636] Letmark P Dahl A (2020) Det här behöver du veta om det nya Coronaviruset. Dagens Nyheter, 28 February, 6.

[bibr42-01634437221147636] Ludvigsson M (2020) I det här läget bör vi följa britterna. Svenska Dagbladet, 18 March, 2.

[bibr43-01634437221147636] Magnusson J Eliasson H (2020) Faran inte över när vaccinering startar. Göteborgs-Posten, 22 December, 5.

[bibr44-01634437221147636] Manzoor A (2020a) Nu måste vi alla bidra för att minska effekterna av pandemin. Dagens Nyheter, 12 March, 8.

[bibr45-01634437221147636] Manzoor A (2020b) Detta vet experterna efter ett halvår med viruset. Dagens Nyheter, 30 June, 6.

[bibr46-01634437221147636] Manzoor A (2020c) Vi får nog vänja oss vid lokala utbrott. Dagens Nyheter, 27 June, 4.

[bibr47-01634437221147636] Martikainen J Sakki I (2021) How newspaper images position different groups of people in relation to the COVID-19 pandemic: A social representations approach. Journal of Community & Applied Social Psychology 31(4): 465–494. 33821119 10.1002/casp.2515 PMC8014754

[bibr48-01634437221147636] Milutinović I (2021) Media framing of COVID-19 pandemic in the transitional regime of Serbia: Exploring discourses and strategies. Media Culture & Society 43(7): 1311–1327.

[bibr49-01634437221147636] Mohammed S Peter E Killackey T , et al. (2021) The “nurse as hero” discourse in the COVID-19 pandemic: A poststructural discourse analysis. International Journal of Nursing Studies 117: 103887. 33556905 10.1016/j.ijnurstu.2021.103887 PMC9749900

[bibr50-01634437221147636] Montessori NM (2016) CDA, critical events and critical event studies: How to make sense of critical events in a society of radical change. In: Lamond RI Platt L (eds) Critical Event Studies. London: Palgrave Macmillan, pp.131–148.

[bibr51-01634437221147636] Nilsson J (2020) Svenskt miljonbidrag för att stoppa viruset. TT Nyhetsbyrån, 24 February, 24.

[bibr52-01634437221147636] Panzeri F Di Paola S Domaneschi F (2021) Does the COVID-19 war metaphor influence reasoning? PLoS One 16(4): 1–20. 10.1371/journal.pone.0250651 PMC8081452 33909699

[bibr53-01634437221147636] Pedersen L (2020) Nu lever vi i pandemins tidevarv. TT Nyhetsbyrån, 11 March, 1.

[bibr54-01634437221147636] Pike KL (1993) Talk, Thought, and Thing, the Emic Road Towards Conscious Knowledge. Dallas, TX: Summer Institute of Linguistics.

[bibr55-01634437221147636] Pirttisalo Sallinen J Kudo P (2020) Sjukvårdsdirektören: Kravet på att ligga steget före har varit stort. Svenska Dagbladet. 9 May, 3.

[bibr56-01634437221147636] Pomerantz A (1986) Extreme case formulations: A way of legitimizing claims. Human Studies 9(2-3): 219–229.

[bibr57-01634437221147636] Potter J (1996) Representing Reality: Discourse, Rhetoric and Social Construction. London: SAGE.

[bibr58-01634437221147636] Potter J Edwards D (1992) Discursive Psychology. London: SAGE.

[bibr59-01634437221147636] Potter J Wetherell M (2001) Unfolding discourse analysis. In: Wetherell M Taylor S Yates SJ (eds) Discourse Theory and Practice: A Reader. London: SAGE, pp.198–209.

[bibr60-01634437221147636] Poulakidakos S (2021) Media events, speech events and propagandistic techniques of legitimation: A multimodal analysis of the Greek Prime Minister Kyriakos Mitsotakis’ public addresses on the SARS-CoV-2 pandemic. Humanities and Social Sciences Communications 8: 237.

[bibr61-01634437221147636] Regeringen (2020) Covid-19 och ändringar i smittskyddslagen. [In English: Government proposition, 2019/20:144]. Regeringen: Stockholm. Available at: https://www.regeringen.se/rattsliga-dokument/proposition/2020/04/prop.-201920144/ (accessed 1 May 2021).

[bibr62-01634437221147636] Reynolds J Wetherell M (2003) The discursive climate of singleness: The consequences for women’s negotiation of a single identity. Feminism & Psychology 13: 489–510.

[bibr63-01634437221147636] Rose N (1999) Powers of Freedom: Reframing Political Thought. Cambridge: Cambridge University Press.

[bibr64-01634437221147636] Sachs L (2020) Vem som ska få leva en svår och ovan fråga. Svenska Dagbladet, 3 April, 41.

[bibr65-01634437221147636] Schegloff EA (1997) Whose text? Whose context? Discourse & Society 8: 165–187.

[bibr66-01634437221147636] Semino E (2008) Metaphor in Discourse. Cambridge: Cambridge University Press.

[bibr67-01634437221147636] Semino E (2021) Not Soldiers but Fire-fighters metaphors and Covid-19. Health Communication 36(1): 50–58. 33167731 10.1080/10410236.2020.1844989

[bibr68-01634437221147636] Silverman D (1993) Interpreting Qualitative Data: Methods for Analyzing Talk, Text and Interaction. London: SAGE.

[bibr69-01634437221147636] Skog F Lundström R (2022) Heroes, victims, and villains in news media narratives about COVID-19. Analysing moralising discourse in Swedish newspaper reporting during the spring of 2020. Social Science & Medicine 294: 114718. 35085897 10.1016/j.socscimed.2022.114718 PMC8743484

[bibr70-01634437221147636] Söderqvist J (2020) Han såg viruset komma - men vi är bara i början. Svenska Dagbladet, 13 May, 20.

[bibr71-01634437221147636] Sontag S (1979) Illness as Metaphors. New York, NY: Farrar, Straus and Giroux.

[bibr72-01634437221147636] Spector M Kitsuse JI (2009) Constructing Social Problems. New York, NY: Aldine de Gruyter.

[bibr73-01634437221147636] Van Dijk TA (1988) News as Discourse. Hillsdale, NJ: Erlbaum.

[bibr74-01634437221147636] Van Dijk TA (1998) Opinions and ideologies in the press. In: Bell A Garrett P (eds) Approaches to Media Discourse. Oxford: Blackwell, pp.21–63.

[bibr75-01634437221147636] Vasterman PL Ruigrok N (2013) Pandemic alarm in the Dutch media: Media coverage of the 2009 influenza A (H1N1) pandemic and the role of the expert sources. European Journal of Communication 28(4): 436–453.

[bibr76-01634437221147636] Von Hall G (2020) Varnar ledarna: “Liknande virus kan återkomma inom tre- fyra år”. Svenska Dagbladet, 20 June, 10.

[bibr77-01634437221147636] Von Hall G Widell L (2020) Därför blir viruset kvar i fem år - medan vi får “nya normala liv”. Svenska Dagbladet, 3 May, 14.

[bibr78-01634437221147636] Wager E Kleinert S (2011) Responsible research publication: International standards for authors. A position statement developed at the 2nd World Conference on Research Integrity, Singapore, July 22–24, 2010. In: Mayer T Steneck N (eds) Promoting Research Integrity in a Global Environment. Singapore: World Scientific, pp.309–316

[bibr79-01634437221147636] Wallis P Nerlich B (2005) Disease metaphors in new epidemics: The UK media framing of the 2003 SARS epidemic. Social Science & Medicine 60(11): 2629–2639. 15814187 10.1016/j.socscimed.2004.11.031 PMC7117051

[bibr80-01634437221147636] Wetherell M (1998) Positioning and interpretative repertoires: Conversation analysis and poststructuralism in dialogue. Discourse & Society 9: 387–412.

[bibr81-01634437221147636] WHO (2020) Director-General’s opening remarks at the media briefing on COVID-19. 11 March 2020. Available at https://www.who.int/director-general/speeches/detail/who-directorgeneral-s-opening-remarks-at-the-media-briefing-on-covid-19—11-march-2020 (accessed 5 May 2021).

[bibr82-01634437221147636] Wolodarski P (2020) Sverige betalar ett högt pris för grupptänkande och hybris. Dagens Nyheter, 29 November, 4.

